# Language and Race in Europe

**Published:** 1894-12-08

**Authors:** 


### THE STUDY OF MAN.

### IX.—LANGUAGE AND RACE IN EUROPE.

In a recent article an outline was given of the method by which it has been believed possible to infer, from similarities of language, a historical blood-relationship between different peoples. But it was intimated that this method is subject to the very serious drawback, that it may always have happened that one people has adopted the language of another race, with which it has no natural kinship, and forgotten its own.

The problem may be set forth more clearly by means of an instance. It is now more than half a century since, on linguistic evidence, a very ancient connection was accepted as existing between all the great races of Europe, and between this group and the Armenians, Persians, and Hindoos; and a number of genealogical trees of the so-called Aryan races have been drawn up to illustrate this connection, agreeing in many important respects, but differing widely in details. The earlier of them agreed in asserting that the "home" of the Aryans was in Central Asia, and that the different Aryan-speaking clans migrated thence in successive hordes to the south and west.

It became, however, difficult to explain why the many Aryans of the West should have travelled so much further from home, than the comparatively few southern stems; and Central Europe has been vigorously claimed, on a number of grounds, as a rival "home," with the Asiatic Aryans as the most distant emigrants. Still more recently Eastern Europe has been proposed as a compromise between these extremes, and, in particular, the upper and middle valley of the Volga. The Southern Steppes, moreover, present the conditions which approximate most nearly to those of the Aryan "home," as it is reconstructed from the linguistic evidence.

Examination of the human remains preserved in early graves, barrows, cave refuges, and pile dwellings, and comparison of these with the types of modern peoples, make us acquainted with four principal and very distinct races of man in Europe. Their characters and geographical range are as follows:—

A. *Iberian.*—Of short stature, with dark complexion, upright face and long narrow skull (index 70—74), limited now to North-West Scotland, West Ireland, South Wales, North Spain, Corsica, and South Italy; but found in early tombs, such as the "long barrows" of England, the caves of Central France, Gibraltar, and the Canaries, and in primitive settlements in Greece and the Troad. Their nearest living counterparts are the Berbers of North Africa, and it is probable that the race spread from the South-west of Europe northwards.

B. *Teutonic.*—Tall, blue-eyed, fair-haired, and fully-bearded; also with long narrow skulls (68—72), but with projecting jaws, low retreating forehead, and small cranial capacity. Best represented in Scandinavia, where the type is perhaps indigenous, but traceable from the Netherlands, up the Rhine, into West Switzerland, and as far east as Bohemia; also in graves throughout Pomerania, and as far east as St. Petersburg. The historical incursions of Germani, Alemanni, Franks, and Normans are referable to this race, which gives its character still to the North-west of Germany.

C. *Ligurian.*—Short and dark, with a very broad skull (85—90), wide across the temples and narrowing below; strikingly like that of the Lapps. Represented throughout Central and Southern France, in Savoy and the Riviera, and in caves as far as Leghorn; also in East Switzerland, beyond the route of the Keltic migrations southward. South-west France presents a mixed race, with Ligurian traits superimposed upon the older Iberian.

D. *Keltic or Central European.*—Tall and ruddy; skull short and broad (80—81), best represented now by the Slavs, the Danes, and the ruddy Scotch and Irish. There is evidence that this race flooded Central Europe in "Neolithic" times, and overflowed thence by Switzerland into Italy, through the Balkan Peninsula into Greece, and through Belgium into the British Isles, driving before it, or subjecting, at all points the Iberian tribes. It occupied north and central France, under the name of Galli or Belgae, and threatened Italy and Greece again in historical times; but the Vosges and Alps formed a natural shelter for the Ligurian race.

Now which of these physical races, if any, can be regarded as representing the Aryan stock? Not the Iberians, with their African affinities and decadent position, in face of the more civilised races from the East. Not the Ligurians, whose area is bounded on the west by the Alps, the Vosges, and the Ardennes, and whose nearest kinsmen are the "non-Aryan" Lapps of the far North. The choice is between the fair long-headed Scandinavians and Teutons from the North, and the ruddy broad-headed Kelts and Slavs from the East. Nor is there even here any obvious correspondence with the genealogical conceptions of philology. The physical character of the Asiatic Aryans does not help us to decide; but the evidence of the early migrations, so far as they can be traced by their remains, is somewhat in favour of the Keltic race. This stock has preserved its greatest purity in Central and Eastern Europe, nearest, that is, to the probable linguistic "home." Its offshoots have carried typical Aryan languages, and the great central-European culture, into the peninsular of Greece and Italy—possibly even into Asia Minor, into northern France, and across the Channel into our own country.

It has lost, it is true, many of its outward characteristics in these further regions by intermixture with the aboriginal peoples, for the primæval races, unless absolutely exterminated, seldom fail to re-assert themselves in course of time, and obliterate the physiognomy of the new comers among their descendants. Witness the steady disappearance of fair hair among the old Greeks, the mediæval Italians, and the modern Germans. But, while it is the blood of the aborigine, it is the speech of the more civilised which survives, though the very changes which have occurred in Aryan languages are just such as always occur when the less civilised people acquires the language of its conquerors. Aryan words are mispronounced, terminations are lost, constructions are misapplied, the older race communicates a distinct accent or idiom to the new language, while even such descendants as survive of the invading "Aryans," lose their individuality and become merged, like our own Saxons, Danes, and Normans, in the mass of the new nation.

There is, indeed, an "Aryan family," but it is a family of foster-brothers.

